# Missense Variant in MAPK Inactivator *PTPN5* Is Associated with Decreased Severity of Post-Burn Hypertrophic Scarring

**DOI:** 10.1371/journal.pone.0149206

**Published:** 2016-02-12

**Authors:** Ravi F. Sood, Saman Arbabi, Shari Honari, Nicole S. Gibran

**Affiliations:** Department of Surgery, UW Medicine Regional Burn Center, Harborview Medical Center, Seattle, WA, United States of America; Sudbury Regional Hospital, CANADA

## Abstract

**Background:**

Hypertrophic scarring (HTS) is hypothesized to have a genetic mechanism, yet its genetic determinants are largely unknown. The mitogen-activated protein kinase (MAPK) pathways are important mediators of inflammatory signaling, and experimental evidence implicates MAPKs in HTS formation. We hypothesized that single-nucleotide polymorphisms (SNPs) in MAPK-pathway genes would be associated with severity of post-burn HTS.

**Methods:**

We analyzed data from a prospective-cohort genome-wide association study of post-burn HTS. We included subjects with deep-partial-thickness burns admitted to our center who provided blood for genotyping and had at least one Vancouver Scar Scale (VSS) assessment. After adjusting for HTS risk factors and population stratification, we tested MAPK-pathway gene SNPs for association with the four VSS variables in a joint regression model. In addition to individual-SNP analysis, we performed gene-based association testing.

**Results:**

Our study population consisted of 538 adults (median age 40 years) who were predominantly White (76%) males (71%) admitted to our center from 2007–2014 with small-to-moderate-sized burns (median burn size 6% total body surface area). Of 2,146 SNPs tested, a rare missense variant in the *PTPN5* gene (rs56234898; minor allele frequency 1.5%) was significantly associated with decreased severity of post-burn HTS (*P* = 1.3×10^−6^). In gene-based analysis, *PTPN5* (*P* = 1.2×10^−5^) showed a significant association and *BDNF* (*P* = 9.5×10^−4^) a borderline-significant association with HTS severity.

**Conclusions:**

We report *PTPN5* as a novel genetic locus associated with HTS severity. PTPN5 is a MAPK inhibitor expressed in neurons, suggesting a potential role for neurotrophic factors and neuroinflammatory signaling in HTS pathophysiology.

## Introduction

During the past 75 years, advances in modern burn care have led to dramatic improvement in the survival of patients with large burns [1]. Unfortunately, most of these survivors develop hypertrophic scarring (HTS), resulting in red, raised, stiff, contracted and often painful and pruritic scars that are both highly disfiguring and disabling [2]. Current strategies for preventing and treating HTS, including pressure garments, topical silicone, and surgical excision, are largely unchanged in the past half-century and yet still lack evidence to support their efficacy [3]. Thus, a great need exists for a better understanding of HTS risk factors and biology in order to develop better therapies for HTS.

Current understanding of HTS biology implicates inflammation and mechanical tension as two key features of the local wound environment that drive HTS formation [4, 5]. Cutaneous injury leads to activation of the clotting and complement cascades and platelet aggregation, which promote the release of chemokines and cytokines that recruit and activate a variety of inflammatory cells such as neutrophils, macrophages, mast cells, T-lymphocytes [5]. These cells in turn produce cytokines including transforming growth factor beta 1 (TGF-β1) that stimulate fibroblast differentiation into myofibroblasts, which contract wounds and deposit extracellular matrix (ECM) and thus have a central role in HTS formation [6]. In addition to secreted signaling molecules, the mechanical microenvironment profoundly influences myofibroblast function, with increased mechanical stress promoting myofibroblastic differentiation and enhanced wound contraction [4].

The mitogen-activated protein kinases (MAPKs) are highly conserved mediators of intracellular responses to both chemical and mechanical extracellular stimuli. They comprise a large interactive network sharing many upstream kinases and downstream effector molecules [7]. Three main classes of MAPKs in humans include the extracellular signal-regulated kinases (ERKs), c-Jun N-terminal kinases (JNKs), and p38 kinases, each of which is rapidly activated by sequential phosphorylation steps by specific upstream MAPK kinases [8]. Activated MAPKs phosphorylate downstream targets including other protein kinases, nuclear proteins, and/or transcription factors and are involved in regulating a range of inflammatory cellular responses including proliferation, differentiation, and apoptosis [9], processes known to be dysregulated in cells contributing to post-burn HTS [5]. Indeed, It has been shown that burn injury activates p38 MAPK signaling in rats, and inhibition of p38 MAPK decreases expression of pro-inflammatory molecules and reduces burn-induced apoptosis of hair-follicle cells [10]. In addition, p38 signaling is known to mediate mechanical-stretch-induced expression of fibrogenic molecules alpha smooth muscle actin (α-SMA) and TGF-β1 in fibroblasts [11]. In accordance with these findings, inhibition of p38 MAPK decreases fibroblast contractility *in vitro* and attenuates wound contraction *in vivo*, both in a rat model [12] and in the red Duroc pig [13], a breed genetically predisposed to form thick, contracted scars closely resembling human hypertrophic scars [14].

Despite progress in our understanding of cellular and molecular events leading to HTS, what triggers these events to different degrees in different individuals remains elusive. Although physiologic (e.g., age, sex) and wound-specific (e.g., burn size, depth, and number of operations) factors are known to be associated with risk of HTS [15], our recent observations that race is independently associated with HTS severity strongly implies a genetic mechanism [16, 17]. However, to date there are few published studies on genetic factors influencing post-burn HTS [16–19], and thus the genetic determinants of HTS remain largely unknown. Given the known role of MAPKs in HTS biology, we hypothesized that single-nucleotide polymorphisms (SNPs) in MAPK-pathway genes would be associated with severity of HTS following burns.

## Methods

### Study design, population, and setting

The University of Washington Institutional Review Board approved this study, and all subjects provided informed, written consent. In addition, we obtained a National Institutes of Health Federal Certificate of Confidentiality. We analyzed data from our recently published prospective-cohort genome-wide association study (GWAS) [19]. We enrolled adults (age ≥18 years) admitted to our institution with burns that were at least deep-partial-thickness or with delayed healing (≥2 weeks), placing them at increased risk of HTS [15]. Subjects provided blood samples for genotyping and were followed in clinic for scar assessment using the Vancouver Scar Scale (VSS; Table 1) [20]. Subjects with at least one completed scar assessment and genotypic data passing quality-control were included in the analysis.

**Table 1:** The Vancouver Scar Scale [20].

Pigmentation*	
Normal	0
Hypopigmentation	1
Hyperpigmentation	2
Vascularity	
Normal	0
Pink	1
Red	2
Purple	3
Pliability	
Normal	0
Supple	1
Yielding	2
Firm	3
Banding	4
Contracture	5
Height	
Normal (flat)	0
>0 and <2 mm	1
≥2 and <5 mm	2
≥5 mm	3

*Since hyperpigmentation is not necessarily more severe than hypopigmentation, this nominal categorical variable was recoded as binary (1 point for either hypo- or hyperpigmentation) for regression analysis.

### Genotyping and quality control

Genotyping and quality control methods were performed as previously described [19]. Briefly, genomic DNA was purified from a venous blood sample and genotyped at the University of Washington Center for Clinical Genomics on HumanCoreExome-12 v1.1 BeadChips (Illumina, San Diego, CA) containing 542,585 genome-wide markers. Although this array genotypes only a fraction of the estimated ten million SNPs common in humans [21], it includes a combination of tag SNPs and exome-focused markers to optimize both indirect genomic coverage and ability to directly identify possible functional variants. We excluded samples with ≥3% missing genotypes and markers with minor allele frequency (MAF) <1%, ≥10% missingness, or Hardy-Weinberg disequilibrium *P*<1×10^−6^.

### Exposure- and outcome definitions

Our exposures of interest were single-nucleotide polymorphisms (SNPs) within MAPK-pathway genes, which we defined as the 267 genes listed in the Kyoto Encyclopedia of Genes and Genomes (KEGG) [22] MAPK signaling pathway. Whereas in our previous genome-wide analysis we used VSS height score alone as our outcome [19], in this study we used the four VSS variables (Table 1) simultaneously as a joint outcome for two reasons: 1) we hypothesized that MAPK-pathway genes contribute to HTS severity by altering the inflammatory response to injury, and inflammation may influence cutaneous angiogenesis [23] and/or pigmentation [24] without concomitant fibrosis; and 2) joint analysis of multiple correlated phenotypes confers increased statistical power both by taking advantage of information provided by cross-phenotype covariance and by avoiding the increased multiple-testing burden associated with separate analysis of multiple phenotypes [25]. For subjects with more than one scar assessment, the highest score for each VSS variable was used.

### Statistical analysis

For descriptive purposes, categorical variables were summarized as number (percent), and continuous variables as median (interquartile range [IQR]). For SNP-based genetic association testing, we used MultiPhen [26] to fit inverted linear regression models of SNP genotype on the four VSS variables, which were coded as shown in Table 1, with the exception of the pigmentation variable. Because the VSS pigmentation variable is scored on a nominal rather than ordinal scale (i.e., hyperpigmentation is not necessarily more severe or adverse than hypopigmentation), the pigmentation variable was re-coded for regression analysis as 0 for normal and 1 for either hypo- or hyperpigmentation. SNP genotypes were modeled as continuous, assuming an additive model of inheritance. In order to adjust for population stratification, we included the first two global principal components as covariates [27]. In addition, we also adjusted for the following factors previously associated with risk of HTS [15]: age (continuous), sex (binary), percent total body surface area (%TBSA) burned (continuous), and number of operations (continuous). The likelihood ratio test was used to obtain a single joint *P*-value for the association between SNP genotype and the four VSS variables simultaneously.

In a secondary analysis, we used the GATES (Gene-based Association Test using Extended Simes procedure) algorithm to perform gene-based association analysis [28]. This powerful approach combines individual-SNP *P*-values to obtain a single *P* value for each gene while controlling the type I error rate regardless of the number of typed SNPs per gene or linkage disequilibrium between SNPs. In order to capture regulatory regions and neighboring SNPs in linkage disequilibrium, we defined gene boundaries as 5 kb upstream and downstream of the 5’ and 3’ untranslated regions (UTRs), respectively. We used data from the 1000 genomes project [29] to account for linkage disequilibrium between SNPs. In both SNP- and gene-based analyses, we used Bonferroni correction to simply and conservatively account for multiple testing; for ease of interpretation, we adjusted significance thresholds and report unadjusted *P*-values. Quality control and principal component analysis were performed using PLINK [30] version 1.90, gene-based association testing was performed using KGG (Knowledge-based mining system for Genome-wide Genetic studies) version 3.5 [28], and all other analyses were performed in the R software environment [31] version 3.1.2.

## Results

**Fig 1:**
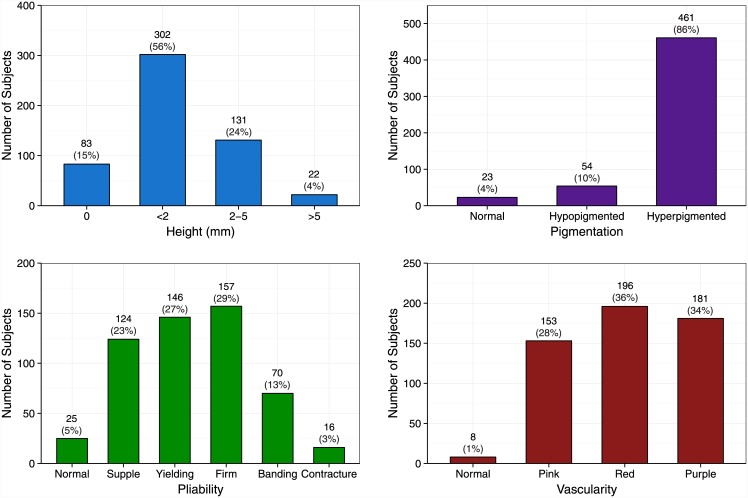
Distribution of Vancouver Scar Scale scores for the 538 study subjects. Data labels are number (percent). Percentages may not sum to 100 due to rounding error.

Over 8 years, we enrolled 638 subjects, 25 of whom were lost to follow-up prior to either providing a blood sample or having a scar assessment. Of the 613 genotyped, 15 subjects had genotypic data that did not pass quality control (≥3% missing genotypes), and 60 were either lost to follow-up or had not yet had a scar assessment at the time of our analysis. The 538 subjects included in our analysis were predominantly young non-Hispanic White males (Table 2), consistent with our burn center’s patient demographics. Given that we enrolled subjects based on having deep and/or slow-healing wounds, most required at least one operation despite having generally small-to-moderate-sized (i.e., <20% TBSA) burns (Table 2). In addition, most subjects developed severe scarring, as illustrated by their high VSS scores (Fig 1).

**Table 2:** Characteristics* of 538 subjects.

Age^#^ (years)	40	(28–53)
Sex		
Male	382	(71%)
Female	156	(29%)
Ethnicity^†^		
Hispanic	72	(13%)
Non-Hispanic	448	(83%)
Race^‡^		
White	408	(76%)
Asian	26	(5%)
Black/AA	19	(4%)
Native American	11	(2%)
Other/multiple	57	(11%)
Burn size^#^ (%TBSA)	6	(2–14)
Number of operations		
None	201	(37%)
At Least One	337	(63%)

*Data presented as number (%), except where indicated.

^#^Reported as median (interquartile range).

^†^Missing or reported as unknown for 18 subjects.

^‡^Missing or reported as unknown for 17 subjects. AA, African American; %TBSA, percent total body surface area burned.

**Fig 2:**
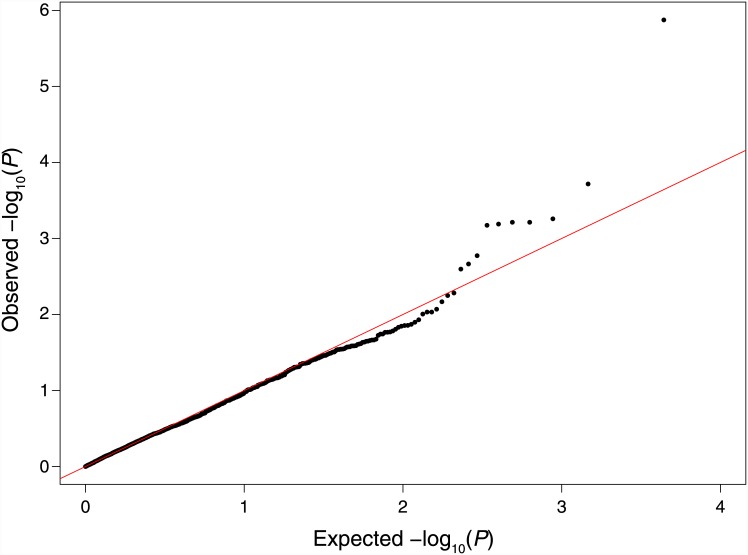
Quantile-quantile plot of *P* values from association testing of MAPK-pathway gene SNPs with severity of post-burn scarring. The solid line indicates the expected null distribution.

Of the 267 genes in the KEGG MAPK pathway, our genotyping array contained 4,912 SNPs in 264 genes. After quality-control filtering, 2,146 unique SNPs in 219 MAPK pathway genes remained for further analysis (S1 File). Testing each SNP for association with the four VSS variables simultaneously in a joint, inverted regression model with adjustment for population stratification by principal component analysis (S1 Table) yielded a *P*-value distribution that did not deviate considerably from the expected null distribution (Fig 2). Furthermore, the genomic inflation factor was estimated to be 1.00 (95% CI: 0.99–1.01), indicating that there was no evidence of test-statistic inflation due to residual confounding by population stratification in our genetically admixed cohort.

**Fig 3:**
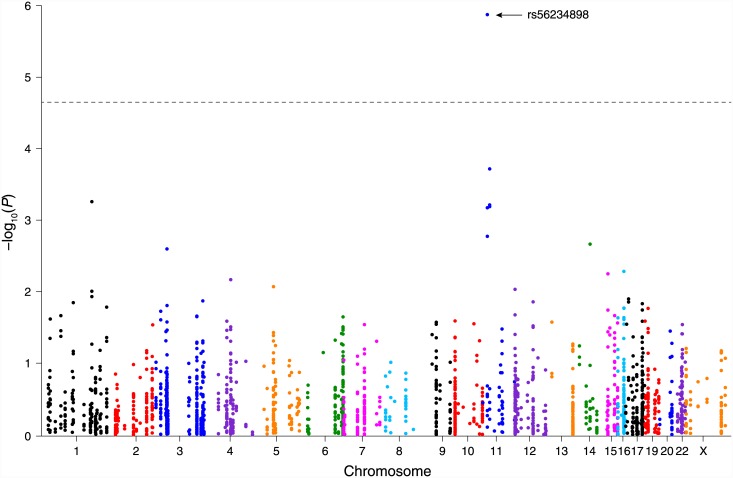
Manhattan plot of *P* values for MAPK-pathway SNP association testing with severity of post-burn scarring. The dashed line corresponds to the analysis-wide significance threshold (*P* = 2×10^−5^). No chromosome 18 or 21 SNPs are shown because none of the KEGG MAPK pathway genes are located on those chromosomes.

Using a Bonferroni-adjusted *P*-value significance threshold of 0.05/2146 ≈ 2×10^−5^, we identified one SNP, rs56234898, that was significantly associated with scar severity (*P* = 1.3×10^−6^; Fig 3). This rare (MAF 1.5% in our study population) exon variant in the protein tyrosine phosphatase, non-receptor type 5 (*PTPN5*) gene causes the substitution of proline for serine at residue 66. It was associated with abnormal scar pigmentation (estimated regression coefficient *β* = 0.206) but with decreased VSS height (*β* = −0.0019), pliability (*β* = −0.0073), and vascularity (*β* = −0.0057) scores. Thus, the missense variant rs56234898 was associated with overall decreased scar severity after adjusting for age, sex, burn size, number of operations, and population stratification.

In a secondary analysis, we used the individual-SNP *P*-values to perform gene-based association testing. Mapping SNPs to genes and excluding non-MAPK-pathway genes yielded 219 genes for analysis (S2 Table). Using a Bonferroni-adjusted *P*-value significance threshold of 0.05/219 ≈ 2×10^−4^, *PTPN5* was significantly associated with HTS severity (*P* = 1.2×10^−5^). This association was based on *P*-values for 10 SNPs located within the *PTPN5* gene. The *BDNF* gene was also strongly associated with HTS severity (*P* = 9.5×10^−4^), based on 7 SNPs within the gene. This association approached but did not quite reach statistical significance after conservative Bonferroni correction for multiple testing.

## Discussion

The clinical observation that people of color seem predisposed to HTS has long implied a genetic mechanism [32], yet until recently, studies of genetic risk factors for HTS have been scarce. Analyzing data from an ongoing prospective cohort study, we recently identified a SNP in the melanocortin 1 receptor (*MC1R*) gene that was strongly associated with increased risk of severe HTS [16]. A subsequent genome-wide analysis identified a SNP in the CUB and Sushi Multiple Domains 1 (*CSMD1*) gene associated with decreased severity of post-burn HTS [19]. Here, taking a candidate-gene approach based on the hypothesis that early wound inflammation contributes to HTS, we have identified the *PTPN5* gene as an additional risk-associated locus. In addition, *BDNF* had a borderline-significant association with HTS severity. Due to the potential for false-positive findings in genetic-association studies, replication of our findings will be critical prior to clinical translation [33], where they might ultimately be useful for predicting scar outcomes and guiding preventive strategies. In addition, investigating the biological mechanisms linking *PTPN5* and *BDNF* to HTS may improve our understanding of HTS pathogenesis.

PTPN5, more commonly referred to as STEP (for STriatal-Enriched protein tyrosine Phosphatase), is a protein tyrosine phosphatase that contains a 16-amino-acid kinase-interaction motif (KIM), which binds specifically to MAPKs [34]. STEP preferentially binds and inactivates ERK1/2 and p38 over JNK [35] and is expressed in the central nervous system, where it has been extensively studied in regulation of dopaminergic and glutaminergic neurotransmission and synaptic strengthening [36]. STEP has been shown to be overactive in neuropsychiatric disorders including Alzheimer’s disease, schizophrenia, and fragile X, and there has been interest in developing STEP inhibitors as to be used as therapies for these disorders [37].

In the present study, the *PTPN5* SNP rs56234898 was associated with decreased HTS severity. Although this SNP may simply be in linkage disequilibrium with one or more true causal variants, it causes an amino-acid substitution, raising the question of a direct effect on STEP protein function as a potential mechanism influencing HTS. Whereas MAPK activation is regulated at the protein level by phosphorylation, phosphatases are regulated at the transcriptional level, and thus it might be expected that genetic differences affecting MAPK regulation would more likely modify phosphatase activity. Given that STEP is a known inhibitor of p38 and that p38 inhibition leads to decreased fibrogenesis experimentally [11, 12], rs56234898 could decrease scarring severity through enhanced STEP-mediated p38 inhibition in myofibroblasts or other cutaneous or inflammatory cells involved in HTS. Although STEP expression has generally been regarded as CNS-specific [37], it has also been suggested that STEP may be expressed in peripheral neurons [38]. Since, to the authors’ knowledge, there are no previous studies linking STEP to wound healing, studying the expression and localization of STEP during cutaneous wound repair will thus be important prior to more in-depth investigation of the potential function of STEP in post-burn scarring. For instance, the red Duroc pig has recently emerged as a promising model for the study of fibroproliferative scarring; this breed is genetically predisposed to form thick scars similar to human hypertrophic scars, in contrast to the Yorkshire pig, which heals normally and serves as a same-species control [14]. Characterizing differences in *PTPN5* polymorphisms and STEP function between red Durocs and Yorkshires may help to elucidate its potential role in scar pathophysiology.

In our gene-based association analysis, *BDNF* was strongly associated with severity of HTS. Although this association fell short of reaching analysis-wide significance after conservatively accounting for multiple testing, it suggests a potential role for *BDNF* in HTS. The BDNF protein binds tropomyosin-related kinase B (TrkB), a membrane-bound receptor tyrosine kinase, which triggers at least three signaling cascades, including downstream MAPK activation [39]. Like STEP, BDNF is expressed in the central nervous system and has been implicated in a variety of neuropsychiatric disorders, including major depressive disorder, bipolar disorder, and schizophrenia [40]. These similarities parallel the result of our recent genome-wide analysis, which linked a common *CSMD1* SNP to decreased HTS severity [19]. Similar to both STEP and BDNF, CSMD1 is expressed in neurons, specifically in growth cones of regenerating neuronal axons [41]; it is implicated in neuropsychiatric disorders [42]; and it is known to regulate inflammatory processes (complement activation [43] and TGF-β1/SMAD signaling [44]). We have previously reported that hypertrophic-scar samples contain increased numbers of nerve fibers [45] as well as elevated levels of substance P [46], a neuroinflammatory mediator released from peripheral nerve fibers after injury, leading us to hypothesize that dysregulated inflammatory signaling by regenerating nerve fibers might have a role in HTS pathophysiology [47]. That STEP, BDNF, and CSMD1 are all expressed in neurons and involved in inflammatory signaling-pathways lends further support to this hypothesis. In addition, BDNF is known to mediate nerve regeneration after injury [48, 49] and was recently found to be over-expressed by inherently fibrogenic dermal fibroblasts isolated from the red Duroc pig [50]. Thus, results of our genetic association analyses support further investigation into the role of nerves and neurotrophic factors in cutaneous scarring.

Limitations of this study include the fact that subject enrollment was constrained by patient demographics of our burn center, and our study population consisted mostly of self-identified Whites, the racial group at lowest risk of severe HTS [16]. As a result, we were unable to detect variants potentially explaining increased incidence and severity of HTS in dark-skinned populations. Further studies will be needed to identify associated genetic variants in high-risk racial groups. In addition, with fewer than 600 subjects included in the analysis, we were at risk of missing true associations due to lack of statistical power given the multiple-testing burden associated with testing thousands of SNPs. We thus sought to increase the power of our analysis through joint association testing with the four VSS variables, which did allow us to detect an association with a relatively rare (MAF 1.5%) variant. We conclude that joint analysis of multiple scar-related outcomes as implemented in packages such as MultiPhen [26] is well suited to genetic association studies of HTS, in which there are multiple relevant scar-related outcomes. However, the somewhat increased complexity of our analysis could potentially challenge its reproducibility. As mentioned previously, our results await confirmation in an independent clinical cohort, since the presently analyzed dataset is to our knowledge the only one available that contains both genotypic and scar-outcome data for hundreds of burn patients. In order to facilitate reproducibility of our analysis, we have used standard scar-outcome measures and have taken care to describe our analytic approach in detail.

In summary, through a candidate-gene approach, we have identified *PTPN5* as a novel genetic locus associated with HTS severity. In addition, our results are suggestive of an association with *BDNF*. Both of these genes are involved in MAPK signaling, are expressed in neurons, and have previously been implicated in the pathogenesis of neuropsychiatric disorders. These findings as well as those from our recent genome-wide association study suggest a potential role for neurotrophic factors and neuroinflammatory signaling in HTS pathophysiology. Our results support further genetic association studies both to confirm our findings and to identify additional genetic variants associated with severity of HTS after burns.

## Supporting Information

S1 File Genetic dataset used to perform the SNP-based association analysis. Genotype data are supplied in PLINK [30] binary format, and phenotype- and covariate data are provided as a tab-delimited text file.
(ZIP)

S1 Table Individual SNP-based testing of 2,146 SNPs in MAPK-pathway genes for association with HTS severity. Results are from joint testing of all four VSS variables using MultiPhen [26], with adjustment for age, sex, %TBSA burned, number of operations, and population stratification.
(XLS)

S2 Table Gene-based testing of 219 MAPK-pathway genes for association with HTS severity using the GATES method [28]. (XLS)
